# Management of Aggressive Acral Melanoma in a Pregnant Patient

**DOI:** 10.7759/cureus.85840

**Published:** 2025-06-12

**Authors:** Leonard R Maier, Maria J Maier, Sharath Rajagopalan, David Shi, Vikash Kumar

**Affiliations:** 1 Internal Medicine, West Virginia University, Morgantown, USA; 2 Medicine, Marshall University Joan C. Edwards School of Medicine, Huntington, USA

**Keywords:** acral melanoma, fungating mass, maternal cancer, maternal-fetal medicine, metastatic melanoma, oncology in pregnancy, pregnancy

## Abstract

Acral melanoma is a rare and aggressive subtype of melanoma that arises on non-sun-exposed areas such as the soles, palms, and nail beds. It is often diagnosed at advanced stages and carries a poor prognosis. We present the case of a 28-year-old pregnant woman at 17 weeks gestation who developed a rapidly enlarging, ulcerated mass on the plantar aspect of her left foot. Biopsies confirmed acral lentiginous melanoma with extensive metastatic spread to the bones, liver, spleen, and lymph nodes. Immunohistochemical analysis was positive for MART-1, preferentially expressed antigen in melanoma (PRAME), and SOX10. After multidisciplinary counseling, the patient elected to terminate her pregnancy to initiate systemic immunotherapy. Despite treatment, her disease progressed rapidly, and she passed away shortly after the initiation of therapy. This case highlights the complexities of diagnosing and managing advanced melanoma in pregnancy and underscores the importance of the early recognition of atypical acral lesions, the limitations of treatment options during gestation, and the ethical challenges in balancing maternal and fetal health.

## Introduction

Acral melanoma represents a rare yet aggressive subtype of cutaneous melanoma, comprising approximately 2%-3% of melanomas in individuals of Caucasian descent and exhibiting a disproportionately higher incidence among individuals with darker skin types. Unlike the more prevalent superficial spreading melanoma, a subtype of melanoma, acral melanoma emerges in non-sun-exposed regions, such as the soles of the feet, the palms, and the nail beds. It is not associated with exposure to ultraviolet (UV) radiation. Due to its unusual location and frequently non-pigmented appearance, it is often misidentified as benign lesions, including warts, ulcers, or skin changes related to trauma, consequently contributing to delays in diagnosis and a poor prognosis at the time of presentation.

Diagnosis is generally established during advanced stages, and the prognosis is markedly unfavorable in comparison to superficial spreading melanoma. A substantial study involving U.S. patients indicated that the five-year survival rate for acral melanoma was lower than that of other melanoma subtypes, particularly when diagnosed at later stages [1]. Furthermore, acral melanoma is characterized by a more biologically aggressive nature, presenting deeper Breslow thickness and elevated mitotic rates at the time of diagnosis [2].

Although rare, melanoma during pregnancy poses distinct and intricate challenges in clinical management. It is the most prevalent cancer that can spread to the placenta and fetus, and pregnancy may lead to alterations in maternal immunity and hormonal balance that could potentially influence tumor behavior, though current data remain inconclusive. Treatment choices must weigh maternal prognosis against fetal safety, particularly since systemic treatments like immune checkpoint inhibitors are not recommended during pregnancy due to potential risks of teratogenicity and immunologic consequences for the fetus [3,4].

This case report describes a young woman who presented during the second trimester of pregnancy with advanced, widely metastatic acral melanoma. The case highlights both the aggressive nature of this melanoma subtype and the ethical and medical complexities involved in managing metastatic cancer during pregnancy.

## Case presentation

A 28-year-old woman, G8P1061, presented at 17 weeks and four days gestation with a painful, enlarging mass on the plantar aspect of her left foot. She was transferred to our tertiary care center for further evaluation after concern for malignancy was raised at an outside facility. Her past medical history included asthma, hypertension, and anxiety. Her obstetric history was notable for five prior spontaneous abortions and a stillbirth at 17 weeks gestation, with no documented underlying cause.

**Figure 1:**
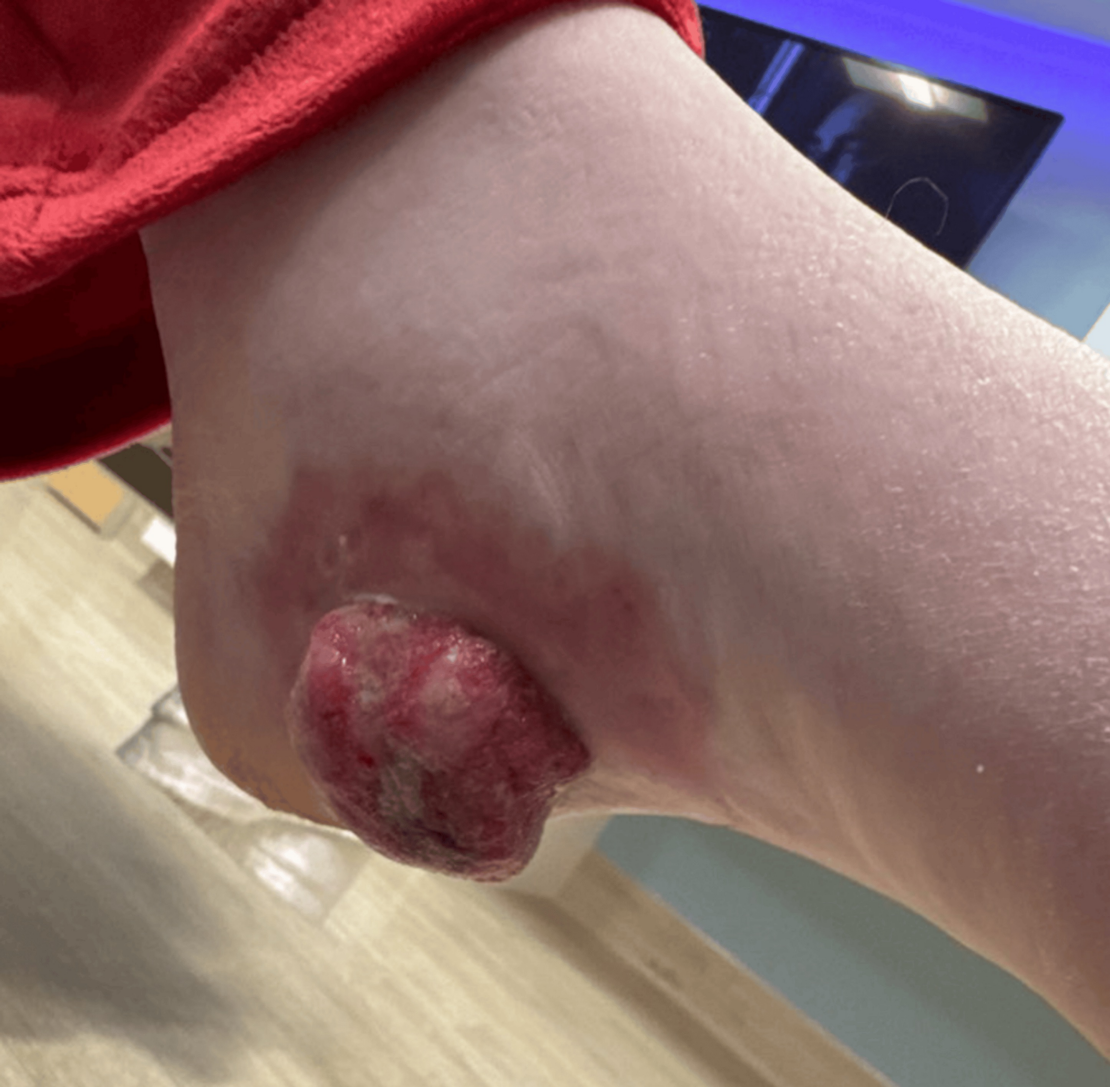
Clinical image of the primary acral melanoma. Photograph of the patient's left foot demonstrating a large, ulcerated, and fungating mass over the plantar heel. The lesion appeared erythematous with irregular borders, necrotic regions, and serosanguinous drainage. This lesion was biopsied and confirmed to be acral lentiginous melanoma, representing the primary site of malignancy.

The lesion was first noticed as a scab on her heel nearly a year prior, rapidly progressing in size and ulceration during pregnancy. At the time of admission, the mass was necrotic, fungating, and measured approximately 6 cm in diameter (Figure 1). The patient also reported fatigue and unintentional weight loss exceeding 20 pounds over the prior year.

**Figure 2:**
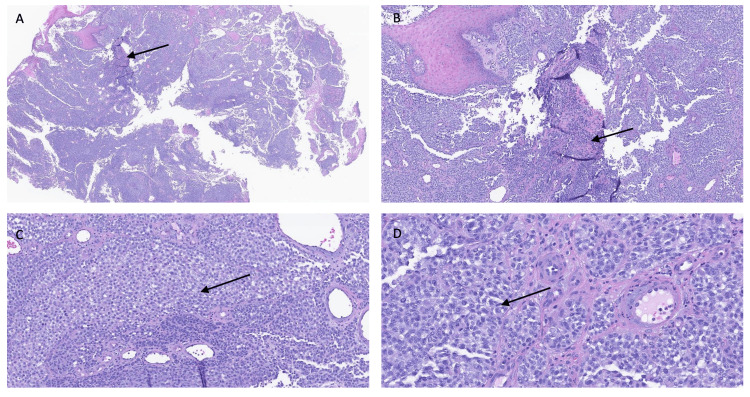
Histopathologic evaluation of the primary acral melanoma. Hematoxylin and eosin (H&E)-stained sections of the primary foot lesion shown at increasing magnification (A–D). The tumor demonstrates markedly atypical, infiltrative melanocytic cells with abnormal mitotic figures. Neoplastic cells involve all surgical margins of the submitted tissue. Features are consistent with invasive acral lentiginous melanoma.

**Figure 3:**
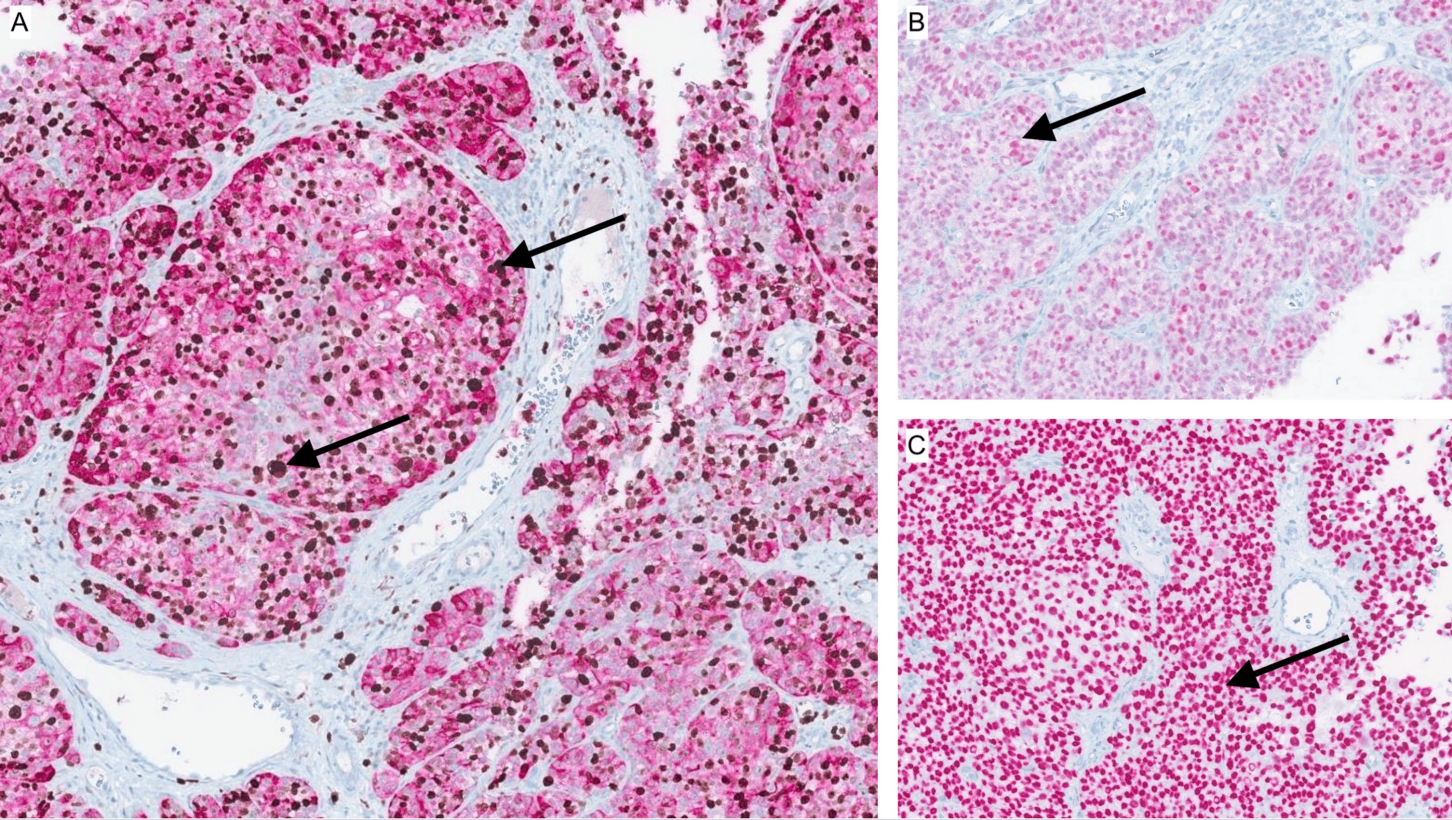
Immunohistochemical staining of the primary melanoma. Panels (A-C) show immunohistochemical stains of the primary acral melanoma. (A) MART-1 stain demonstrating diffuse strong cytoplasmic positivity in lesional melanocytic cells. (B) PRAME stain showing nuclear positivity, supporting a malignant phenotype. (C) SOX10 stain highlighting nuclear expression, confirming melanocytic lineage. Together, these markers support the diagnosis of malignant melanoma. PRAME: preferentially expressed antigen in melanoma

**Figure 4:**
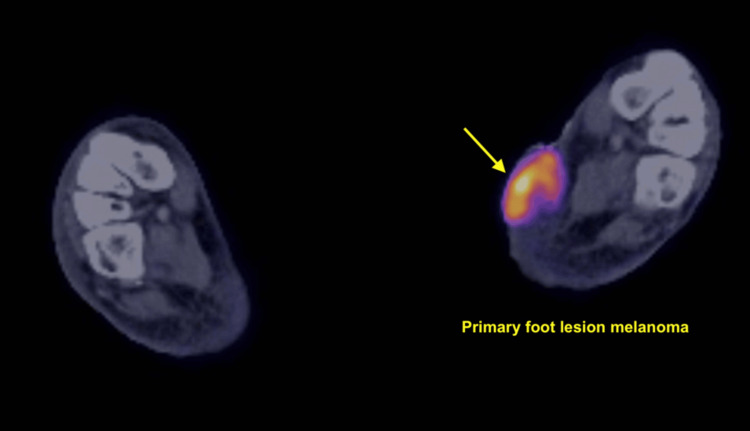
PET-CT imaging of the bilateral feet. Axial fused PET-CT image showing intense FDG uptake (yellow arrow) in a soft tissue lesion located on the plantar surface of the left foot, corresponding to the primary acral melanoma. The lesion is hypermetabolic, consistent with high tumor activity. This served as the primary site of malignancy in the patient. FDG: fluorodeoxyglucose

**Figure 5:**
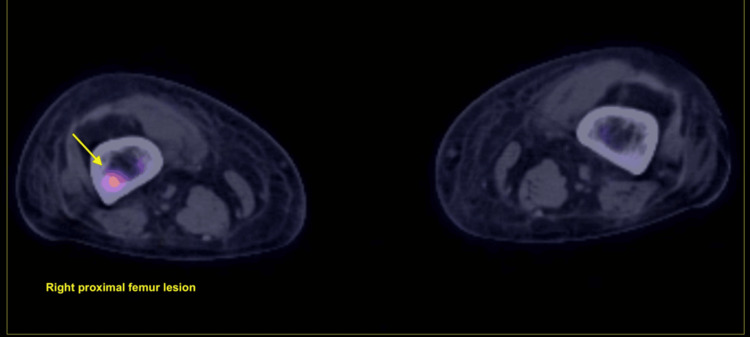
PET-CT imaging of the bilateral proximal femurs. Axial fused PET-CT image demonstrating intense FDG uptake (yellow arrow) in the right proximal femur, consistent with a hypermetabolic osseous metastatic lesion. The contralateral femur (right side of the image) shows no abnormal uptake. This lesion represents part of the patient's extensive skeletal metastatic burden from acral melanoma. FDG: fluorodeoxyglucose

**Figure 6:**
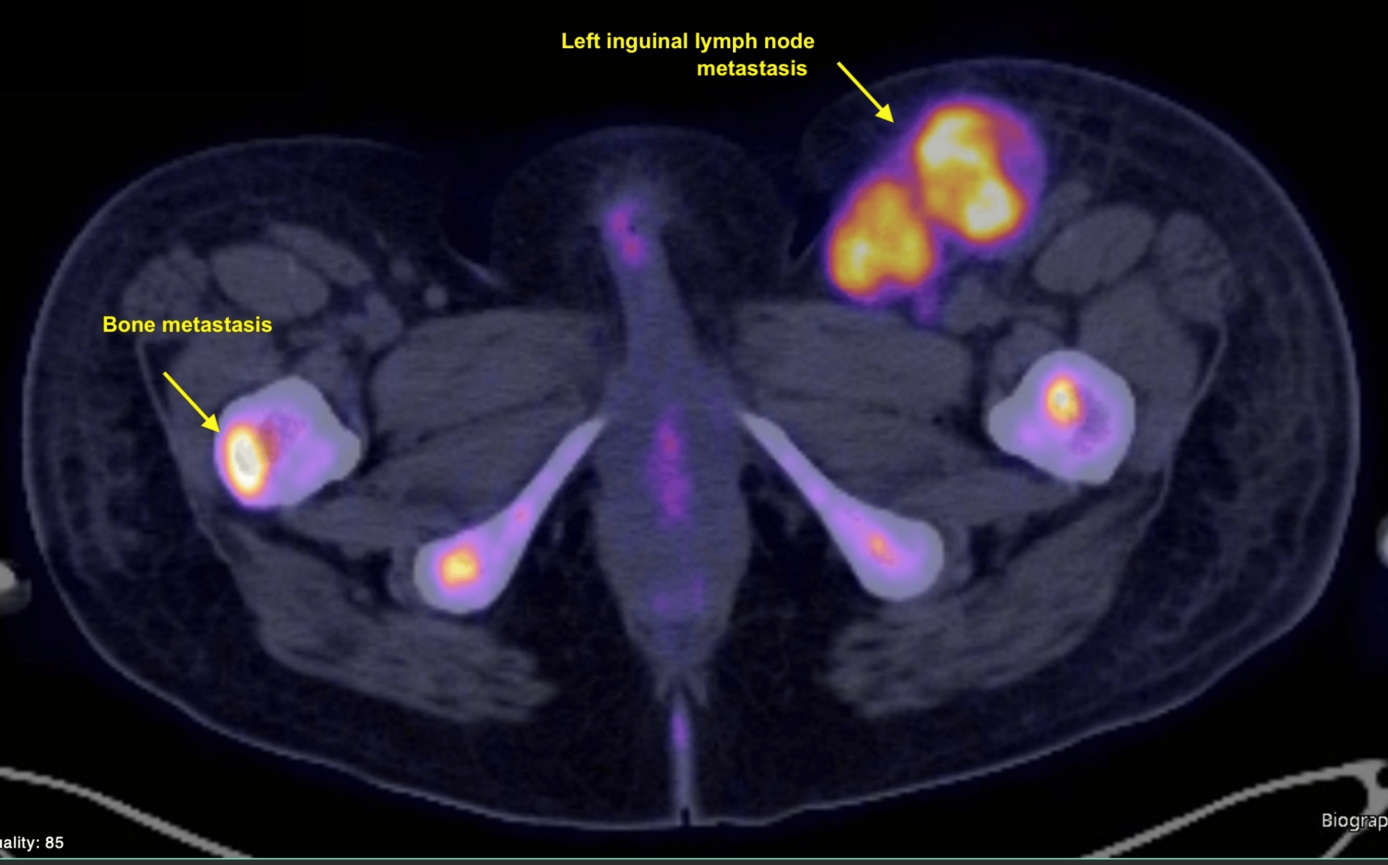
PET-CT imaging of the pelvis. Axial fused PET-CT image demonstrating intense FDG uptake in a large left inguinal lymph node (yellow arrow, upper right), measuring 3.6 cm in the short axis with SUV max 13.1, and a focal hypermetabolic lesion in the left proximal femur (yellow arrow, lower left), consistent with osseous metastasis. These lesions were identified as index lesions for treatment response assessment in a patient with widely metastatic acral melanoma. FDG: fluorodeoxyglucose; SUV: standardized uptake value

**Figure 7:**
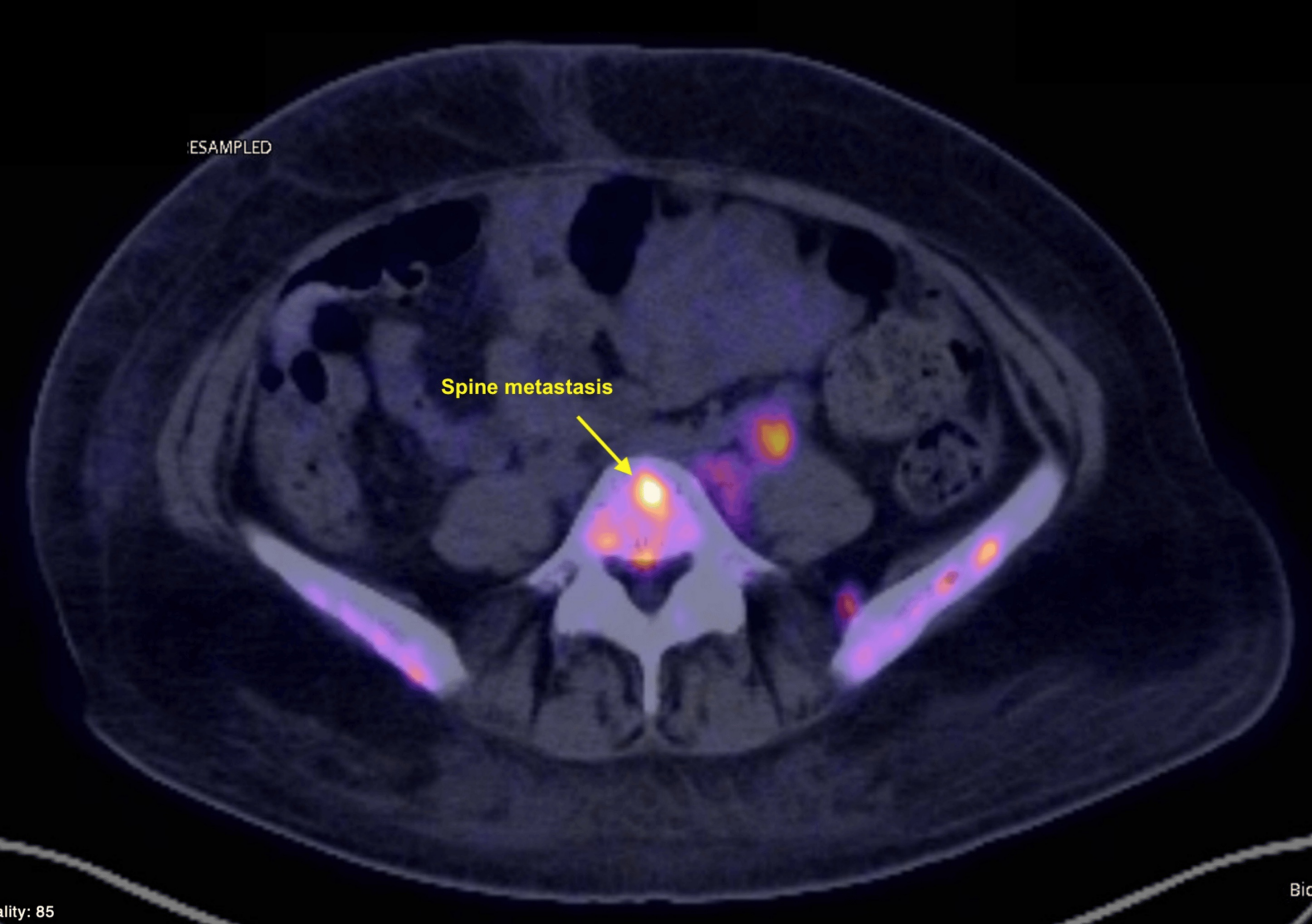
PET-CT imaging of the lumbar spine. Axial fused PET-CT image showing focal hypermetabolic activity in a lumbar vertebral body (yellow arrow), consistent with osseous metastasis to the spine. This lesion was one of several sites of skeletal involvement in this patient with widely metastatic acral melanoma.

**Figure 8:**
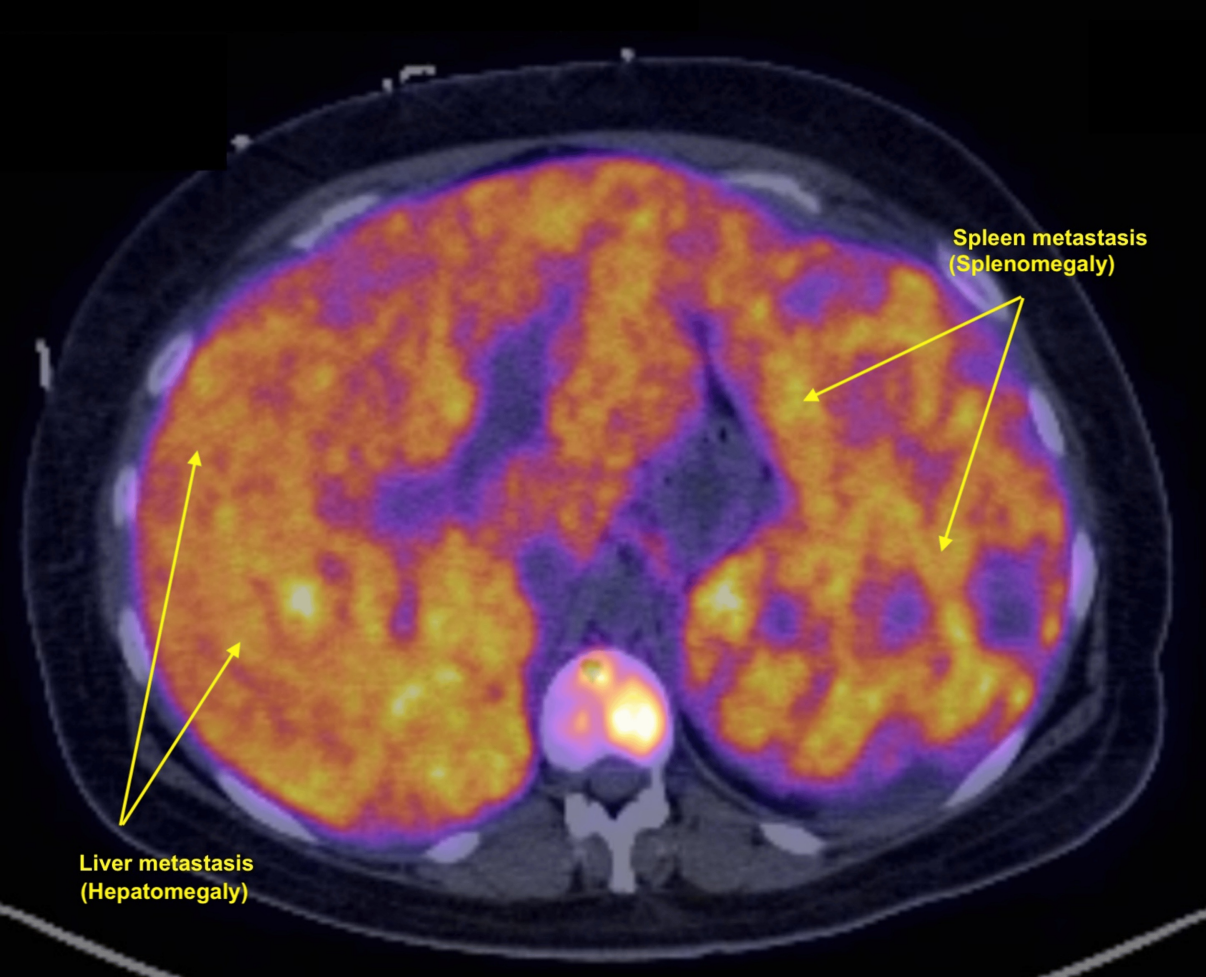
PET-CT imaging of the upper abdomen. Axial fused PET-CT image demonstrating diffuse, heterogeneous FDG uptake throughout the liver and spleen, consistent with widespread hepatic and splenic metastases (yellow labels). The pattern of hypermetabolism in both organs reflects extensive tumor infiltration in the patient with metastatic acral melanoma. FDG: fluorodeoxyglucose

Initial diagnostic workup included a bedside punch biopsy of the foot lesion and an interventional radiology-guided core biopsy of an enlarged left inguinal lymph node. Histopathology revealed malignant melanoma of acral origin, with immunohistochemical staining positive for MART-1, preferentially expressed antigen in melanoma (PRAME), and SOX10 (Figures 2, 3). PET-CT imaging showed extensive hypermetabolic lesions consistent with widespread metastatic disease, involving nearly all visualized bones (including the spine and femurs), the liver, the spleen, pelvic and retroperitoneal lymph nodes, and the left foot (Figures 4-8). MRI of the brain revealed no evidence of intracranial metastases.

The case was reviewed during an extended multidisciplinary meeting with input from hematology-oncology, maternal-fetal medicine, obstetrics, palliative care, supportive care, and psychological support. The patient’s complex medical and psychosocial circumstances were discussed at length, with emphasis on prognosis, pregnancy-related considerations, and treatment goals. Given the advanced stage and aggressive biology of the disease, systemic therapy was urgently recommended. However, available immunotherapeutic options, including PD-1 and CTLA-4 inhibitors, are contraindicated in pregnancy due to potential fetal harm. After in-depth counseling, the patient elected to undergo induction of labor at 18 weeks gestation to allow for timely treatment.

Following pregnancy termination, she received outpatient checkpoint inhibitor-based immunotherapy. Despite brief periods of clinical stabilization, her disease progressed rapidly. She developed increasing pain and functional decline and required ongoing blood transfusions for anemia of chronic disease. Despite the best supportive care and immunotherapy, the patient ultimately succumbed to complications of an extensive metastatic burden and passed just a few weeks later.

## Discussion

Acral melanoma is a unique subtype of cutaneous melanoma, notable for its non-sun-exposed locations, late detection, and significantly poorer prognosis. It usually develops on the palms, soles, or nail beds, where initial lesions may be misidentified as benign issues like warts, ulcers, or changes due to trauma. This often causes delays in diagnosis. In contrast to superficial spreading melanoma, acral melanoma tends to be diagnosed at more advanced stages, frequently showing greater Breslow thickness and a higher mitotic index, which results in a markedly lower five-year survival rate, especially in metastatic situations. A comprehensive population-based study by Bradford et al. found that acral lentiginous melanoma had poorer outcomes compared to other melanoma types, even when accounting for the diagnosis stage [1].

Immunohistochemical analysis played a key role in confirming the diagnosis of malignant melanoma in this patient. MART-1, a melanocytic differentiation antigen, demonstrated diffuse cytoplasmic positivity, confirming melanocytic lineage. SOX10, a nuclear transcription factor, further supported the diagnosis with strong nuclear staining of melanocytic cells. Most notably, the tumor was positive for PRAME, a tumor-associated nuclear antigen overexpressed in melanoma but typically absent in benign nevi. The combined staining pattern helped distinguish the lesion from benign melanocytic proliferations and supported the diagnosis of acral lentiginous melanoma with malignant features.

This case demonstrates the aggressive progression of acral melanoma, which manifested during the second trimester of pregnancy with extensive metastases upon diagnosis. Similar to the findings reported by Phan et al., our patient exhibited a widespread disease burden, consistent with the typical advanced stage at which acral melanoma is identified [2]. Even though the foot lesion was present before pregnancy, her disease notably progressed despite her relatively young age of onset. This highlights the significance of early biopsy of evolving acral lesions, both during and outside the context of pregnancy.

The overlap of pregnancy and cancer presents distinct ethical and medical dilemmas. Melanoma ranks among the most commonly diagnosed cancers during pregnancy and is the leading type that can metastasize to the placenta or fetus [3,4]. Immunotherapy, especially PD-1 inhibitors and CTLA-4 inhibitors, is now fundamental in treating metastatic melanoma, enhancing survival rates even in advanced stages. Nonetheless, these treatments are contraindicated during pregnancy due to potential risks such as miscarriage, growth restriction in utero, congenital defects, and immune-related adverse effects on both the fetus and the placenta [4-6]. In this case, the patient chose to terminate her pregnancy to begin systemic therapy, highlighting the challenging decisions faced when weighing maternal survival against fetal viability. Although a few case studies report patients postponing treatment until after childbirth, those who delay systemic therapy often face disease progression and poorer outcomes [3]. In some reported cases, transplacental metastasis to the fetus or placenta has also been documented, though this remains rare [4].

This case highlights the significant yet often overlooked function of non-invasive prenatal testing (NIPT) in identifying potential maternal cancers. While NIPT mainly targets fetal chromosomal abnormalities, instances of discordant or multiple aneuploidies, as observed in our patient’s screening, including monosomy 13, monosomy 18, and trisomy X, raise suspicion for maternal neoplasm, a phenomenon increasingly recognized with the widespread use of cell-free DNA screening [7]. It is important for providers to recognize this occurrence and contemplate additional maternal evaluations when patterns such as these present themselves.

Multidisciplinary care played a vital role in aiding our patient with her diagnosis, oncological planning, and decision-making. The cooperation among maternal-fetal medicine, oncology, palliative care, and social support services enabled shared decision-making that resonated with the patient’s values and clinical situation. The patient chose to terminate the pregnancy, focusing on her survival and the need for timely treatment.

## Conclusions

This case highlights the aggressive characteristics and diagnostic difficulties of metastatic acral melanoma, especially when it occurs during pregnancy. Delays in diagnosis and the common presentation at later stages lead to a poor prognosis for this melanoma subtype. The physiological and ethical challenges of pregnancy further complicate management. Early identification of atypical lesions and prompt biopsy are essential, especially during pregnancy. A collaborative approach that includes oncology, obstetrics, maternal-fetal medicine, and palliative support was critical in guiding patient-centered decision-making in this case. This shows that incidental findings on NIPT may serve as early indicators of maternal malignancy and warrant further evaluation when chromosomal abnormalities are discordant or unexplained.

In conclusion, this case shows the importance of timely diagnosis, interdisciplinary coordination, and individualized care planning in achieving the best possible outcomes for pregnant patients with aggressive malignancy. Increased awareness and reporting of similar cases may help refine future guidelines for managing melanoma during pregnancy.
